# Knowledge of Hospice Care among Korean American Immigrants in Deep South

**DOI:** 10.1093/geroni/igab046.2858

**Published:** 2021-12-17

**Authors:** Eunyoung Choi, Hee Lee, Hyunjin Noh, Lewis Lee

**Affiliations:** 1 University of Southern California, LA, California, United States; 2 Univeristy of Alabama, Tuacaloosa, Alabama, United States; 3 University of Alabama, Tuscaloosa, Alabama, United States; 4 Univeristy of Alabama, Tuscaloosa, Alabama, United States

## Abstract

Despite the benefits of hospice care in end-of-life care, there is a dearth of research on the knowledge or perceptions of hospice care, particularly among immigrants. A handful number of existing studies with this population have mainly used qualitative research methods. The purpose of the current study was to investigate the knowledge about hospice care and identify its predictors. We used cross-sectional data from 256 Korean American immigrants living in Alabama (Mean age = 44.78, range 23–70, 50.4% female). The outcome variable was measured by whether the respondents had heard of hospice care. Independent variables included sociodemographic (age, gender, education, and income), health (functional limitation and chronic conditions), health care access (health literacy, health insurance, unmet medical needs due to the cost, and social isolation). Logistic regression analyses were performed. About 78% of the respondents reported that they had heard of hospice care. Older age (OR=1.05, 95% CI=1.01-1.09, p <.05), being female (OR=7.13, 95% CI=3.18-15.98, p <.001), and higher levels of education (OR=1.68, 95% CI=1.15-2.45) were significantly related to increased odds of knowledge about hospice care. There were no significant roles of health and health care access factors. Our findings suggest sociodemographic gradients present in immigrants’ knowledge about hospice care, emphasizing the need for a targeted intervention to increase the hospice care knowledge.

